# Activation of CB2R by synthetic CB2R agonist, PM289, improves brain endothelial barrier properties, decreases inflammatory response and enhances endothelial repair

**DOI:** 10.1515/nipt-2023-0016

**Published:** 2023-10-16

**Authors:** Trent A. Bullock, Kalpani N. Udeni Galpayage Dona, Jonathan F. Hale, Paula Morales, Nadine Jagerovic, Allison M. Andrews, Servio H. Ramirez

**Affiliations:** Department of Pathology and Laboratory Medicine, Lewis Katz School of Medicine at Temple University, Philadelphia, PA, USA; Center for Substance Abuse Research, Lewis Katz School of Medicine at Temple University, Philadelphia, PA, USA; Shriner’s Hospital for Children, Philadelphia, PA, USA; Medicinal Chemistry Institute, Spanish National Research Council, Madrid, Spain

**Keywords:** blood–brain barrier, CB2R agonist, neuroinflammation, endothelial cells, traumatic brain injury, cannabinoid receptor 2

## Abstract

The Cannabinoid 2 Receptor (CB2R) has been found to provide immunological modulation in different cell types. More recently, detection of CB2R in the cerebral endothelium suggests a possible role in the resolution of inflammation at the level of the blood–brain–barrier (BBB). Here, the notion that CB2R upregulation in brain endothelial cells could be exploited to promote vascular protection and BBB integrity was evaluated. Targeting and activation of CB2R was accomplished by a novel and highly specific chromenopyrazole based CB2R agonist, PM289. This study demonstrates that CB2R upregulation is induced as early as 8 h in the cortical vasculature in an experimental mouse model of TBI. Unlike CB2R, CB1R was marginally detected and not significantly induced. In the human brain endothelial cell line, hCMEC/D3 cells, similar induction of CB2R was observed upon stimulation with TNFα. Analysis of transendothelial electrical resistance shows that PM289 markedly prevented the barrier-leakiness induced by TNFα. The BBB is also responsible for maintaining an immunological barrier. The five-fold increase in ICAM1 expression in stimulated endothelial cells was significantly diminished due to CB2R activation. Utilizing wounding assays, results showed that wound repair could be accomplished in nearly half the time when the novel CB2R agonist is present compared to the untreated control. Lastly, mechanistically, the effects of CB2R may be explained by the observed inhibition of the p65 NFκB subunit. Overall, these studies support the notion that targeting and activating CB2R in the brain vasculature could aid in BBB and vascular protection in the context of neuroinflammation.

## Introduction

Numerous studies have demonstrated the involvement of both the Cannabinoid 1 Receptor (CB1R) and Cannabinoid 2 Receptor (CB2R) in the modulation of neuroimmune function [1–3]. Beyond these canonical receptors, a variety of lipid mediators and enzymes (dubbed the endocannabinoidome or eCBDome) have also been identified which may mediate pharmacologic effects of cannabinoids [4]. The psychotropic and potentially addictive effects of CB1R activation in the CNS have limited the use of CB1R targeting compounds [5]. Due to its negligeable level of expression in the CNS, CB2R has historically been referred to as the peripheral cannabinoid receptor [6, 7]. However, in recent years, independent research groups have identified low levels of CB2R expression in neuronal subpopulations and non-neuronal cell types, including brain endothelial cells [8–11].

Brain vascular endothelial cells that line the cerebral vasculature tightly control the solute composition needed for proper neuronal function and are referred to as the blood–brain barrier (BBB) [12]. This restriction of cellular and molecular flux between the vessel lumen and brain parenchyma is due to the intricate complex of tight and adherens junctions and highly regulated molecular transport systems that define the specialized nature of the brain endothelium [13]. Disruption of the BBB is a key aspect of neuroinflammation irrespective of how neuroinflammation was triggered; whether it was caused by neurotrauma, neurodegeneration, or any other neurological condition in which CNS tissue pathology is evident. Thus, an aberrant effect on the BBB places neuronal networks vulnerable to dysfunction [14, 15].

Although CB2R expression under homeostatic conditions is very low in the CNS, recent discoveries have shown that the level of CB2R is markedly increased at the brain endothelium in conditions of neuroinflammation [1, 10, 11, 16]. Moreover, there is evidence that activation of CB2R by synthetic CB2R agonists promote vascular repair in a variety of neuroinflammatory conditions [10, 17], [18], [19], [20]. Despite this promising evidence, the inducibility of CB2R and specific actions of CB2R on the BBB remain poorly understood during neuropathological conditions.

To date, there is a lack of pharmacological treatments designed to specifically promote BBB integrity, vascular repair and attenuate endothelial response to inflammation. Such treatments could greatly prevent the damage to delicate neuronal networks which can be compromised as a result of traumatic brain injury, stroke, infectious diseases of the CNS or neurodegenerative disorders. Experimentally, triggering BBB repair shows clear vascular benefits that results in lowering the risk of neurological sequalae including reduced cerebral edema, attenuated neurodegeneration, and improved neurocognitive function [21, 22].

The research herein aims to further provide scientific evidence that activation of CB2R by the novel and recently developed chromenopyrazole based CB2R agonist, PM289, provides therapeutic benefits to brain endothelial cells that are both BBB protective and anti-inflammatory. Overall, these results suggest that as a target for vascular and BBB protection, leveraging the endocannabinoid system specifically by highly selective and potent CB2R agonists provides a much-needed solution to alleviating the devastating consequences of neuroinflammation.

## Materials and methods

### Reagents and cell culture

All reagents used for cell culture were handled according to sterile technique. The hCMEC/D3 cell line was purchased from Neta Scientific Inc (Hainesport, NJ) and expanded to at least passage 3 before use in experimentation. Recombinant human Tumor Necrosis Factor α (TNFα) was purchased from Peprotech Inc (Cranbury, NJ) and resuspended in nanopure water with 0.1 % Bovine Serum Albumin (BSA) as recommended by the manufacturer. The commercially available CB2R antagonist, SR144528 was purchased from Cayman Chemical Company (Ann Arbor, MI) and resuspended in dimethyl sulfoxide (DMSO), purchased from Millipore Sigma (St. Louis, MO). The novel synthetic CB2R agonist, PM289 (chemical name 7-(1,1-Dimethylheptyl)-2-ethyl-2,4-dihydro-9-methoxy-4,4-dimethylchromeno[4,3-c]pyrazole) was synthesized by Dr. Paula Morales, as previously described [23] in the Jagerovic Laboratory (Instituto de Química Médica, Consejo Superior de Investigaciones Científica, Madrid, Spain). This compound was dissolved first in 100 % DMSO, then diluted in phosphate buffered saline (PBS, Gibco) to a final DMSO concentration of 10 %, and sterile filtered.

To model the BBB *in vitro*, the hCMEC/D3 cell line was used. All experiments were conducted with cells between passage 3–20. Culture conditions were followed as previously described [24]. Briefly, cells were grown on rat tail collagen type 1 (BD Bioscience, San Jose, CA) coated cultureware at 37 °C, 5 % CO_2_, and 90 % humidity. Cells were grown in EBM-2 (Lonza) supplemented with EGM-2 MV SingleQuots (Lonza Group, Morristown, NJ) with 1 % Penicillin/Streptomycin and 1 % Fungizone by volume. Upon reaching confluency, cells were washed with PBS containing Ca2+ and Mg2+ (Thermo Fisher Scientific, Waltham, MA) and growth media was replaced with unsupplemented EBM-2 containing 5 % FBS, 1 % Penicillin/Streptomycin, and 1 % Fungizone. The day before experiments, media was changed to EBM-2 containing 1 % FBS and all treatments were prepared in EBM-2 containing 1 % FBS.

### Experimental traumatic brain injury (TBI) model

For all animal experiments, six-week-old male C57CBL/6 mice were purchased from the Jackson Laboratory (Bar Harbor, ME). Upon arrival to the facility, mice were group housed under a 12/12 h light/dark cycle with *ad libitum* access to food and water. Mice were allowed a seven-day acclimation period to the University Laboratory Animal Research facility at Lewis Katz School of Medicine at Temple University prior to administration of any experimental procedures. All experiments were approved by the IACUC at Temple University and complied with the Guide for the Care and Use of Laboratory Animals (NIH, Publication 865–23).

Experimental moderate traumatic brain injury was modeled using a previously described controlled cortical impact model [25–28]. Briefly, mice were anesthetized with vaporized isoflurane (5 % induction, 2 % maintenance) and secured in a Just for Mouse™ Stereotaxic Frame (Stoelting Co., Wood Dale, IL) under a Zeiss Stemi 2000-C stereomicroscope (Carl Zeiss Microscopy, LLC, Thornwood, NY) equipped with an SCHOTT EasyLED Ringlight (SCHOTT North America Inc., Elmsford, NY). Deep anesthesia was confirmed by failure of the animal to retract their paw in response to a hind limb toe pinch test. Craniectomy was performed by first cutting a triangular skin flap on the dorsal cranium and then using an Ideal Micro-Drill™ (CellPoint Scientific Inc., Gaithersburg, MD) with a 0.5 mm rounded burr to remove a 4 mm bone flap located to the right of the sagittal suture between lambda and bregma. Then, a moderate experimental TBI (4.5 m/s, 2 mm compression depth, 0.5 s dwell time) was administered using a single discharge from an Impact One™ Stereotaxic CCI Instrument (Leica Microsystems, Buffalo Grove, IL) outfitted with an electromagnetically driven piston (2 mm diameter). After injury induced bleeding stopped, the wound was covered with a sterile 6 mm round glass coverslip (ProSciTech Pty Ltd, Kirwan, Australia) adhered to the skull using Vetbond™ tissue adhesive (3 M, St. Paul, MN). Post operatively, mice were individually housed to ensure surgical recovery; they were weighed and monitored for behavioral abnormalities daily until reaching the experimental endpoint of interest. Sham injured control mice were treated identically except for the discharge of the Impact One™ Stereotaxic CCI Instrument. Naïve control mice were individually housed at the same time as experimental mice.

### *In-situ* hybridization by RNAscope

Mice received moderate CCI-TBI or sham surgery as described above. Twenty-four hours post-TBI, mice were anesthetized and transcardially perfused first with >20 mL ice cold PBS, then with >20 mL ice cold paraformaldehyde in PBS. Brains were removed from the skull and post-fixed in paraformaldehyde for 24 h. Fixed brains were dissected into four coronal sections which were subjected to sucrose gradient (10–30 %) prior to embedding and freezing tissue in Optimal Cutting Temperature embedding media (OCT, Agar Scientific). Frozen tissue was sectioned to 15 µm on a cryostat and adhered to Superfrost Plus slides (Fisher Scientific, Hampton, NH). Tissue slides were kept at −80 °C with desiccant until further processing.

On the day of the assay, slides were washed with PBS to remove excess OCT, baked for 30 min at 60 °C, and post-fixed in ice cold paraformaldehyde in PBS for 15 min. Tissue was sequentially dehydrated in increasing concentrations of ethanol (50 , 70, 100, 100 %) for 5 min at each concentration. Slides were then dried for 5 min at room temperature before applying hydrogen peroxide for 10 min at room temperature to block endogenous peroxidase activity. Peroxide was removed and slides were washed with nanopure water before proceeding to target retrieval. All target retrieval reagents were brought to 99 °C prior to adding the tissue slides. First, slides were submerged for 10 s in nanopure water and then immediately transferred to target retrieval reagent for 15 min to achieve mild and standard conditions. Afterward, slides were immediately washed in room temperature nanopure water for 15 s before being placed in 100 % ethanol for 3 min and then baking at 60 °C for 5 min. Slides were allowed to dry at room temperature overnight before continuing the assay.

The next day, protease reagent was applied to the dry tissue slides for 30 min in a humidified 40 °C oven. Slides were washed twice with nanopure water and then proceeded to target probe binding and amplification. The probes (ACD Bio, Newark, CA) used for hybridization were designed specifically for the detection of mRNA transcripts: Cannabinoid Receptor 2 (CNR2) and Platelet endothelial cell adhesion molecule-1 (CD31/PECAM1). Probe Mm-Cnr2-O2 (436,091) had 8ZZ pairs which targeted the region of 506–934 of the CNR2 mRNA transcript variant 1 with NCBI reference sequence, NM_009924.4. Probe Mm-Pecam1-O1 (471481) had 30ZZ pairs that targeted the 881 to 2410 region of PECAM1 mRNA transcript variant 1 with NCBI reference sequence, NM_008816.3. Together, probes were hybridized to tissue samples for 2 h at 40 °C in a humidified oven. Signal was then amplified with RNAscope (ACD Bio, Newark, CA) amplification reagents according to the manufacturer’s protocol. Tissue was stained with sequential application of horseradish peroxidase, fluorophore, and HRP blocker according to the manufacturer’s protocol. Sections were counterstained with DAPI and mounted with Prolong Gold antifade reagent (Thermo Fisher). Sections were visualized and images acquired with an A1R inverted confocal microscope (Nikon Inc, Melville, NY). Image analysis for particle counting was performed with the AIVIA Imaging software (Leica Microsystems, Wetzlar, Germany).

### Isolation of brain microvasculature

Following experimental CCI-TBI, mice were euthanized and brain microvessels were isolated by dextran gradient centrifugation for downstream molecular analysis at selected timepoints. In brief, mice were anesthetized as previously described and transcardially perfused with >20 mL ice-cold PBS. Mice were then decapitated, and their brains removed from the cranium and quickly placed in ice-cold Hanks Balanced Salt Solution (HBSS). Brains were then dissected into 2 mm coronal sections and the section containing the majority of the impact penumbra was selected for further processing. This coronal section was further dissected into four quadrants and microvessels were isolated only from the upper quadrants ipsilateral to the area of impact and its corresponding contralateral control. Tissue from these quadrants was homogenized separately in 100 µL HBSS and then mixed with 1 mL 17.5 % Dextran (MW 60,000–90,000, MP Biomedicals, Solon OH). Samples were then centrifuged at 4400×*g* for 15 min allowing vascular tissue to collect in the bottom of the tubes while myelin, glial, and neuronal tissue remained floating on top. This tissue and excess dextran solution was carefully removed from the samples before resuspending the microvessel pellet in 1 mL HBSS to wash the tissue. Samples were centrifuged for 10 min at 10,000×*g* and then the HBSS carefully aspirated before resuspending the pellet in 1 mL TRIzol (Invitrogen, Thermo Fisher Scientific) for subsequent gene expression analysis.

### XTT cell viability assay

To determine how experimental treatments affected cell viability, hCMEC/D3 cells were plated at 20,000 cell/well on a black, clear bottom 96-well plate and grown to confluency. After reaching confluency, cells were treated as previously described. Saponin detergent (0.1 % solution) was used as a positive control. Twenty-four hours after treatment, a CyQUANT™ XTT Cell Viability assay (Invitrogen, Thermo Fisher Scientific) was performed according to the manufacturer’s protocol. Briefly, 70 µL of XTT (2,3-Bis-(2-Methoxy-4-Nitro-5-Sulfophenyl)-2H-Tetrazolium-5-Carboxanilide) working reagent was added to experimental, positive control, or blank wells containing 100 μL cell culture media. The plate was incubated at 37 °C for 4 h before reading absorbance wavelengths at 450 and 660 nm. Cell viability was determined from specific absorbance values obtained from the following equation: Specific Absorbance=[Abs_450nm_(Test) − Abs_450nm_(Blank)] − Abs_660nm_(Test)]. Viability was normalized to untreated cells and expressed as a percent of untreated cell viability.

### Quantitative reverse transcriptase PCR and droplet digital PCR

To examine gene expression via qRT-PCR, total Ribonucleic acid (RNA) was isolated as previously described [29, 30]. Briefly, 1 mL TRIzol reagent was added to isolated microvessel tissue to dissociate total RNA from the samples. Samples were further processed with the PureLink RNA extraction kit (Invitrogen, Thermo Fisher Scientific) according to the manufacturer’s protocol. cDNA was synthesized with 250 ng RNA in a 20 µL reaction mix using High-Capacity cDNA Reverse Transcriptase kit (Applied Biosystems, Thermo Fisher Scientific). qRT-PCR was performed using Taqman Universal Fast 2× Master Mix (Thermo Fisher Scientific) and FAM-labeled mouse probes for CNR1 (Mm01212171) and CNR2 (Mm02620087). 18S rRNA was used as an internal control. Gene expression was calculated using the 2^−ΔΔCt^ algorithm.

For digital droplet PCR, confluent hCMEC/D3 cell monolayers were treated with 100 ng/mL TNFα for 6 h and washed with PBS. Cells were harvested as a cell pellet and RNA purification was performed using Qiagen RNeasy mini kit (Qiagen Sciences Inc, Germantown, MD) to examine the mRNA concentrations. cDNA was synthesized using the high-capacity cDNA Reverse Transcription kits (Applied Biosystems, Thermo Fisher Scientific). Quantitative Digital PCR was performed using Absolute Q™ DNA Digital PCR Master Mix (Applied Biosystems, Thermo Fisher Scientific) and CNR1 Taqman Probe Human-FAM-MGB (Cat #: Hs01038522_s1) and CNR2 Taqman Probe Human-VIC-MGB (Cat #: Hs05019229_s1). Gene expression level was quantified using QuantStudio Absolute Q Digital PCR software (Applied Biosystems, Thermo Fisher Scientific).

### Trans-endothelial electrical resistance (TEER)

All electric cell-substrate impedance sensing (ECIS) experiments were conducted as previously described [29–31]. Briefly, real-time changes to Transendothelial Electrical Resistance (TEER) were measured and recorded with the ECIS Z-Theta 96-well array station (Applied Biophysics Inc., Troy NY). Impedance, resistance, and capacitance were recorded over a spectrum of frequencies (1,000–64,000 Hz) using the multiple frequency/time (MFT) function. Prior to cell plating, electrode containing 96W20idf plates were conditioned with 10 mM cysteine solution (Applied Biophysics Inc.) to stabilize gold electrodes and coated with rat tail collagen type 1. hCMEC/D3 cells were plated at a density of 20,000 cells/well in EGM-2 MV media with one well left cell-free for modeling purposes. During the growth stage (days 1–3 post plating) cells were maintained in EGM-2 MV. One half of the media was exchanged every 48 h until cells reached confluency and stable barrier formation indicated by stable resistance ≥400 Ohms and capacitance ≤100 nF at 4,000 Hz. Upon reaching confluency, half the media was exchanged for EBM-2 containing 5 % FBS and cells were monitored for continued confluency and barrier functionality (days 3–5 post plating). The day prior to all experiments, half the media was exchanged for EBM-2 containing 1 % FBS. All treatments were prepared in EBM-2 containing 1 % FBS. Experimental treatments were added in four replicate wells and monitored for 48 h after which the experiment was terminated, and all data was normalized to the baseline resistance data collected prior to treatment. Data are reported as percent of baseline resistance.

### Paracellular permeability assay

The paracellular permeability assay was conducted as previously described [30]. Briefly, cells were plated in 200 µL of EGM-2 media at a density of 20,000 cells per well on the apical portion of semi-permeable trans-well inserts (Fisher Scientific, 0.4 µm pore size). These inserts were placed in 24-well plates containing 800 µL EGM-2 media (basal container). During the growth phase (days 1–3 post plating) half the apical media and all the basal media was exchanged every 48 h until cells appeared confluent under a light microscope. After reaching confluency (days 4–5 post plating), half the apical media and all the basal media was exchanged every 48 h for EBM-2 containing 5 % FBS and 1 % penicillin/streptomycin and 1 % fungizone while cells were monitored for continued confluence. The day before treatment (day 6 post plating), half the apical media and all the basal media was exchanged for EBM-2 containing 1 % FBS, and treatments were also prepared in this same media. Cells were treated as indicated (Supplemental Figure 2) for 24 h. Following treatment, media in the basal chamber was replaced and FITC-conjugated dextran (4 kDa, Millipore Sigma) was added to the apical chamber to a final concentration of 2 mg/mL. Plates were then replaced in the tissue culture incubator for 1 h to allow the FITC-dextran to diffuse. After this one-hour period, the transwell inserts were removed and 200 µL aliquots of basal media were loaded in triplicate into a black, clear bottom 96-well plate to read fluorescence (525 nm) using a SpectraMax M5e (Molecular Devices). Data are expressed as a fold change in permeability relative to untreated cells.

### Electrolysis-based wound assay

The ECIS Z-Theta 96-well array station (Applied Biophysics Inc.) is capable of delivering a high electrical pulse to electrodes present in the plate electrode array. For the wounding phase of the assay, this high energy electrical pulse, critically electroporates and eliminates the cells on the electrodes. Following the wounding phase, cells peripheral to the electrode will begin to divide, migrate and fill-in the open space (the healing phase of the assay). The ECIS wounding assay replaces the traditional scratch assay, featuring various advantages over the traditional one. For instance, the ECIS wounding assay provides an analytical measure of cellular migration, precise and automated wounding, and a high degree of reproducibility that also preserves extracellular matrix coating. In typical ECIS measurements of TEER, a low current of less than 1 μA is primarily used. Such low current used for the measure of TEER has no effect on cellular physiology. However, when the wounding protocol is initiated the current is magnified over 1000-fold from 1 μA to 1.4 mA. Keeping the high current active for 20 s causes a massive voltage increase across the affected cell membranes resulting in severe electroporation and electrolysis. Cell death is indicated by a crash in TEER values. The subsequent healing phase is monitored also by TEER representing the cellular migration and proliferation of cells adjacent to the electrodes.

hCMEC/D3 cells were plated as described under the TEER methods. Impedance, resistance and capacitance were recorded over a spectrum of frequencies (1,000–64,000 Hz). Once steady TEER was reached, the cells on top of the printed electrodes were succumbed to the electroporation/electrolysis wounding parameters consisting of a 60,000 Hz for 20 s at 1,400 µA. TEER was continually monitored up to 72 h following wounding.

### Western blot

Relative protein expression in hCMEC/D3 cells was determined by sodium dodecyl sulfate-polyacrylamide gel electrophoresis (SDS-PAGE). Culture conditions and treatments were applied as previously described. Following experimental treatment, cells were washed with ice-cold PBS containing Ca2+ and Mg2+ (Thermo Fisher Scientific) and lysed with ice-cold 1× Radio-immunoprecipitation assay (RIPA) buffer (Millipore Sigma) prepared in PBS containing Ca2+ and Mg2+. Protein concentration was determined with Bicinchoninic acid (BCA) assay (Thermo Fisher Scientific). Samples containing 10 µg total protein were denatured at 95 °C for 5 min and then loaded onto MIDI sized 4–12 % Bis-Tris XT Criterion gels (Bio-Rad Laboratories, Hercules, CA) for electrophoresis. Protein was transferred from the gels to nitrocellulose membranes (Bio-Rad) using the Trans-Blot Turbo (Bio-Rad Laboratories) system. Adequate protein transfer was confirmed with Ponceau-S staining (Fisher Scientific) after which membranes were washed with nanopure water and blocked for 1 h at room temperature in Superblock in PBS (Thermo Fisher Scientific). Blocked membranes were incubated overnight at 4 °C with the following primary antibody solutions prepared in Superblock: Intercellular Adhesion Molecule-1 (ICAM-1) (sc-8439, Santa Cruz Biotech Inc., Dallas, TX) mouse mAb, 1:1000, and β-actin (A5441, Millipore Sigma) mouse mAb, 1:5000. Membranes were then washed with 0.05 % PBS-T and incubated for 1 h at room temperature in secondary antibody solutions containing goat anti-mouse IRDye 800CW in Superblock (1:15,000). Membranes were washed again 0.05 % with PBS-T and imaged with an iBright FL 1500 instrument (Thermo Fisher Scientific). Band intensities were determined using ImageJ software (NIH, Bethesda, MD). Data is presented as relative intensity of ICAM-1 to β-actin and normalized to untreated samples.

### Immunofluorescence assay

Immunofluorescence detection of Zona Occludens-1 (ZO-1) was performed as previously described [31]. Briefly, confluent cell monolayers were washed with PBS and fixed with 4 % paraformaldehyde for 10 min. Cells were then washed with PBS, blocked and permeabilized with 5 % donkey serum and 0.3 % triton-X for 20 min. To visualize the tight junction protein ZO-1, cells were stained with anti-ZO-1 (BD Biosciences, Franklin Lakes, NJ) diluted at 1:100 for 1 h at room temperature. Secondary antibody anti mouse Alexa fluor 488 (Thermo Fisher Scientific) was used at a dilution of 1:500 for 1hr at RT. The cell nuclei were counterstained with DAPI and the monolayer was mounted with prolong antifade reagent. Images were acquired using a Nikon A1R inverted confocal microscope (Nikon Instruments).

### Enzyme linked immunosorbent assay

Nuclear Factor Kappa B (NFκB) p65 subunit activity was determined by TransAM NFκB Activation Assay (Active Motif Inc. Carlsbad, CA) according to the manufacturers protocol. Briefly, hCMEC/D3 cells were cultured and treated as previously described. After treatment, cells were washed with ice-cold PBS containing Ca2+ and Mg2+ (Gibco) and lysed using TransAM lysis buffer (for nuclear fraction). Sample protein concentration was determined with Pierce 660 nm Protein Assay (Thermo Fisher Scientific) and samples were prepared with 10 µg total protein. Equal concentrations of each sample lysate were added to the assay plate in duplicate and absorbance was read at 450 nm with a reference wavelength of 650 nm.

### Statistical analysis

For *in vivo* experiments, all animals were randomly assigned to an injury group and endpoint time (n=3). Tissue was isolated both contralateral and ipsilateral to the craniectomy and the corresponding anatomical location in naïve mice. Gene expression was normalized to naïve mouse tissue based on anatomical location and compared with One-Way ANOVA using Dunnett’s post-hoc test to compare results between injured tissue and naïve controls. Data are expressed as the mean±S.E.M.

All *in vitro* experiments were conducted at least three times and normalized either to baseline data in the case of ECIS experiments or to untreated control cells for all molecular analyses. Group comparisons were made using One-way ANOVA and post-hoc Tukey’s or Dunnett’s test as appropriate. Results are expressed as the mean±S.E.M. All data was analyzed with GraphPad Prism software v9.5.1 (GraphPad, San Diego, CA).

## Results

### CB2R expression is inducible in the cerebral vasculature following experimental TBI

To better understand the inducible temporal dynamics for the canonical cannabinoid receptor subtypes CB1R and CB2R in the brain vasculature, gene expression analysis was performed. At the specified time (as shown in the figure), brains were removed and the cerebral microvessels were harvested from ipsilateral (same side as the injury) and contralateral (non-impacted hemisphere) areas, and the corresponding anatomical location in samples from naïve mice. Then, total RNA was isolated, cDNA synthesized, and qRT-PCR analysis performed. Results are reported as a fold change in CNR1 and CNR2 gene expression relative to anatomically similar tissue from naïve mice.

**Figure 1:**
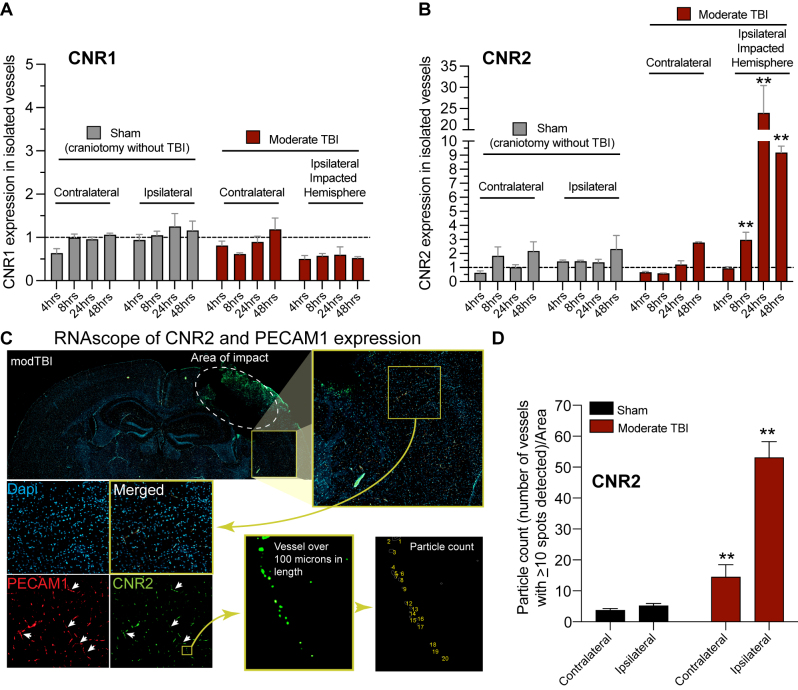
Cannabinoid Receptor 2 (CNR2) expression is upregulated following a traumatic brain injury (TBI). A, B. Microvessels were isolated at 4, 8, 24, 48 h from the contralateral or ipsilateral cortex from sham animals (craniotomy without a TBI) or animals which received a moderate TBI. qPCR was performed to determine the gene expression of the cannabinoid receptor 1 (CNR1) and CNR2. CNR1 levels were unchanged between sham and TBI over time (A). CNR2 expression increased in the area of impact in the TBI animals (B). C. RNA scope was performed to further confirm the increase in CNR2 (Green) was in localized in microvessels. Vessels were identified to be positive for the endothelial marker gene PECAM1 (Red). Dapi (blue) was used to identify cell nuclei. D. Analysis was performed using Aivia software (Leica) to quantify the number of vessels which contained >10 CNR2 spots along a vessel. The ipsilateral side of TBI animals had significantly greater numbers of vessels containing >10 CNR2 spot. All experiments were performed with n=3 animals for each group.

Figure 1, shows the results from a TBI of moderate severity (CCI-TBI) delivered to the cortex of 8 wk old C57BL/6 J mice. Figure 1A, shows the fold changes of CNR1 expression to uninjured controls. Although the overall ANOVA for CNR1 expression ipsilateral to impact was significant [contralateral: (F[8,18]=2.439, p=0.0554); ipsilateral: (F[8,18]=3.745, p<0.01)], Dunnett’s multiple comparisons between naïve samples and injured samples, revealed no significant changes in the inducibility of CNR1 at any of the time points (4, 8, 24 and 48 h) under observation. In addition, no statistically significant changes in CNR1 expression appeared proximal (ipsilateral) or distal (contralateral) to the site of the injury. Unlike CNR1 a different outcome was revealed in the inducibility of CNR2. Figure 1B, provides evidence that CNR2 gene expression is markedly increased at most of the time points (except at 4 h) analyzed [contralateral: (F[8,18]=15.96; ipsilateral: (F[8,18]=11.42, p<0.0001)]. Proximal to the impacted area, CNR2 was upregulated about 3-fold (8 h), 23-fold (24 h) and 9-fold (48 h) post TBI. Of note, only at 48 h was a 2.7-fold increase in CNR2 gene expression observed in the non-impacted hemisphere.

To further confirm that the results in CB2R gene expression are indeed due to endothelial expression and not of those from non-endothelial cells, mRNA *in-situ* hybridization (via RNAscope technology) was utilized. Figure 1C, demonstrates the results from a second cohort of moderate CCI-TBI and sham animals (at 24 h) in which probes specific for CNR2 mRNA were used. In addition, probes for CD31/PECAM1 were also introduced into the tissue sections in order to assure that the CNR2 expression analyzed corresponds to those in the endothelium. As can be seen in Figure 1C a clear detection in CNR2 from increased hybridization of specific fluorescently labeled CNR2 probes can be observed. Acquired images were processed for particle detection and scoring methodology. First, platelet endothelial cell adhesion molecule-1 or CD31/PECAM1 detection identified vascular structures in the tissue which then allowed for the targeting of colocalization of CNR2 signals in such vascular elements. Background correction was applied and vessels of 15 μm in length displaying more than 10 dots was deemed positive for CNR2 increased expression per vessel. Figure 1D, displays the results of the image analysis utilized which shows the number of positive vessels per 4.0 × 10^4^ μm^2^ area.

Taken together the results suggests that inflammation resulting from neurotrauma can significantly induce the expression of CNR2 (CB2R) and not CNR1 (CB1R) thereby implicating the anti-inflammatory CB2R as part of the resolution of inflammatory response at the cerebral vasculature.

### Targeting of CB2R by a novel CB2R synthetic agonist improves BBB integrity

**Figure 2:**
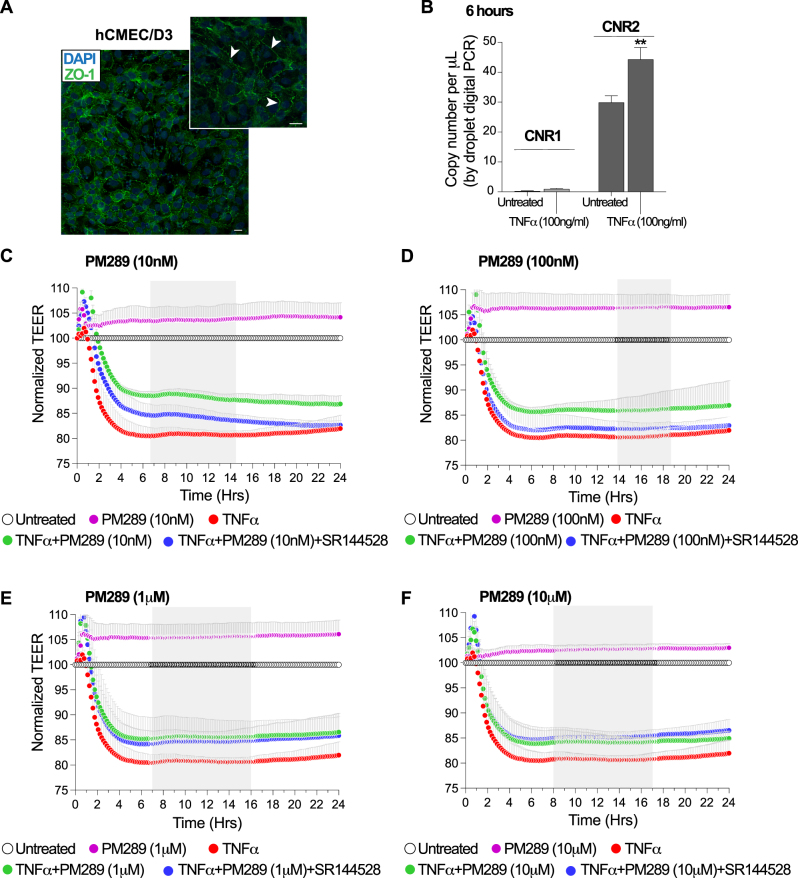
PM289 reduces barrier disruption in brain microvascular endothelial cells. A. hCMEC/D3 cells were examined by immunofluorescence staining for the expression of the tight junction protein zonula occludens 1 (ZO-1). ZO-1 (green) was localized at the cell borders. Dapi (blue) was used to identify cell nuclei. Scalebar equals 10 μm. B. Expression of CNR1 and CNR2 was evaluated by digital PCR in hCMEC/D3 cells in untreated cells and cells treated with 100 ng/mL of TNFα for 6 h. CNR1 expression was very low at baseline and largely unchanged in response to TNFα. Comparatively, CNR2 expression was much higher at baseline and significantly upregulated in response to TNFα. n=3 for each group. C–F. Barrier tightness of hCMEC/D3 monolayers were evaluated by electric cell-substrate impedance sensing (ECIS). Concentrations of PM289 10 nM (C), 100 nM (D), 1 μm (E), and 10 μM (F) were evaluated for 24 h. Data was normalized to the untreated condition (open circles). Groups included PM289 (pink circles), TNFα (red circles), TNFα + PM289 (green circles) and TNF-α + PM289 + SR144 (orange circles). TNF-α significantly decreased barrier tightness which was significantly attenuated at all concentrations of PM289. Additionally, PM289 treatment alone had a barrier tightening effect. All conditions were performed with n=4–8 replicates.

The hCMEC/D3 cerebrovascular endothelial cell line is commonly used as a surrogate for generating a human BBB *in vitro* [32]. hCMEC/D3 cells express endothelial protein markers, tight junction proteins, efflux transporters and other proteins typically seen in human brain endothelial cells. Figure 2A, shows an example of the key tight junction protein, zona occludens 1 or ZO-1 expression in hCMEC/D3 cells after immunostaining. Note the membrane associated compartmentalization of ZO-1 which allows for other proteins to bind to it and form tight junction complexes. To validate that the expression and induced upregulation of CNR2 also applies to human endothelial cells, gene expression assays were performed by droplet digital PCR (dPCR). Digital PCR provides the highest sensitivity possible for determining transgene expression. Figure 2B, shows a marginal (0.3–0.5 copies per μL) detection of CNR1 expression at basal conditions (untreated hCMEC/D3). Stimulation and activation of hCMEC/D3 by 100 ng/mL of TNFα for 6 h shows a slight trend towards an increase in CNR1 but it is not statistically significant. Conversely, analysis of CNR2 shows that hCMEC/D3 cells contain a high level of CNR2 mRNA that can be further enhanced by approximately 15 copies per μL following cytokine exposure.

To test the notion that CB2R expression (particularly inducible expression) in hCMEC/D3 cells can be leveraged for its anti-inflammatory properties, a novel synthetic CB2R agonist, PM289, was used. Barrier tightness by transendothelial electrical resistance (TEER) was explored to evaluate whether CB2R activation can protect against a loss in barrier integrity. After achieving stable baseline TEER readings attributable to monolayer and tight junction formation, the endothelial cells were exposed to the following experimental conditions: untreated, TNFα (100 ng/mL), PM289 (at either 10 nM, 100 nM, 1 μM or 10 μM), TNFα + PM289 and TNFα + PM289 + SR144528. As shown in Figure 2C–F, resistance values consistently dropped (representative of a leaky endothelial barrier) with the introduction of TNFα exhibiting a rapid decrease in resistance that plateaued at ∼20 % below its baseline. Conversely, cells treated concurrently with TNFα and PM289 demonstrated barrier protection by limiting the full extent of severe barrier compromise seen with TNFα alone. Moreover, the response of cells to concurrent treatment with TNFα and PM289 appeared inversely proportional to the dose used (although these statistical comparisons were not performed to simplify analysis). That is, cells treated at the same time with TNFα and 10 nM PM289 exhibited higher normalized TEER values than those treated concurrently with TNFα and 10 µM PM289.

To ensure that the cellular response is CB2R specific, additional experiments were conducted in which hCMEC/D3 cells were also exposed to CB2R antagonist, SR144528 while in the presence of TNFα and PM289. The results indicated that the addition of SR144528 blocked the barrier improving effects of PM289 at the lowest two doses tested (100 and 10 nM), but the cells treated with higher doses of PM289 (10 and 1 µM) exhibited a near identical response. Together, these results suggest PM289 blocks the barrier disruptive effects of TNFα in a dose dependent manner. Of note, addition of PM289 alone (without cytokine activation) provided a stable and sustained strengthening in barrier tightness.

### Anti-inflammatory effect of novel CB2R agonist, PM289 on endothelial activation

**Figure 3:**
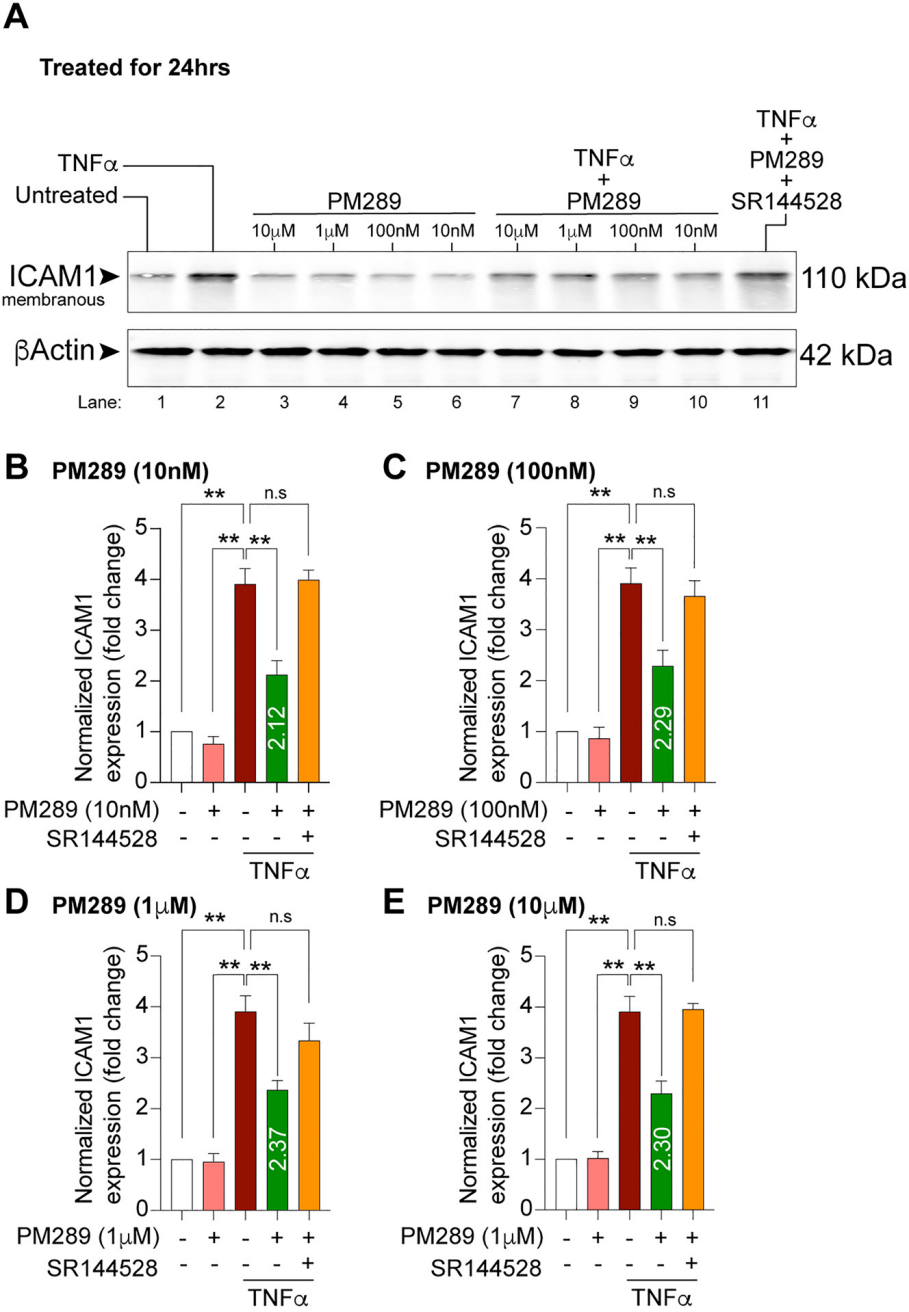
PM289 attenuates TNFα-induced upregulation of ICAM-1. A. hCMEC/D3 cells were treated with 100 ng/mL TNFα for 24 h with or without concentrations with PM289 (10 nM, 100 nM, 1 μM and 10 μM). Controls included untreated cells and cells treated with PM289 only. One group included TNFα, PM289 and SR144528. Cells were harvested in lysis buffer and proteins were separated by SDS-page for Western blot analysis. The adhesion molecule ICAM-1and β-Actin were resolved at 110 and 42 kDa respectively. B–E. Quantification of changes in ICAM-1 expression normalized to the untreated condition for PM289 concentrations 10 nM (B), 100 nM (C), 1 μM (D) and 10 μM (E). For all concentrations PM289 significantly reduced ICAM-1 expression in response to TNFα. Treatment with the CB2R antagonist SR144528 prevented the effects of PM289. All experiments were performed with n=3. **p<0.01.

To test the effects of the novel CB2R agonist, PM289, on the immunological barrier function of brain endothelial cells, experiments were conducted to assess the expression of intercellular adhesion molecule 1 (ICAM1). hCMEC/D3 cells were treated with TNFα (100 ng/mL), PM289 (10 nM–10 µM), SR144528 (10 µM) or combinations thereof. Figure 3A, shows representative images of Western blot results from membranes probed with anti ICAM1 and anti β-actin (loading control) antibodies. Figure 3B–E, shows densitometry results indicating that TNFα induced approximately a 4-fold increase in ICAM1 relative to untreated samples as expected. With the addition of PM289, TNFα treated cells showed a significantly lower ICAM1 expression at each dose tested (F[13,28]=30.47, p<0.0001). This effect appeared to be CB2R specific since addition of SR144528 resulted in ICAM1 levels returning to those observed in cells treated only with TNFα. Together, these data suggest that CB2R activation regulates a key aspect of brain endothelial inflammatory response.

### Brain endothelial CB2R activation accelerates wound recovery and healing

**Figure 4:**
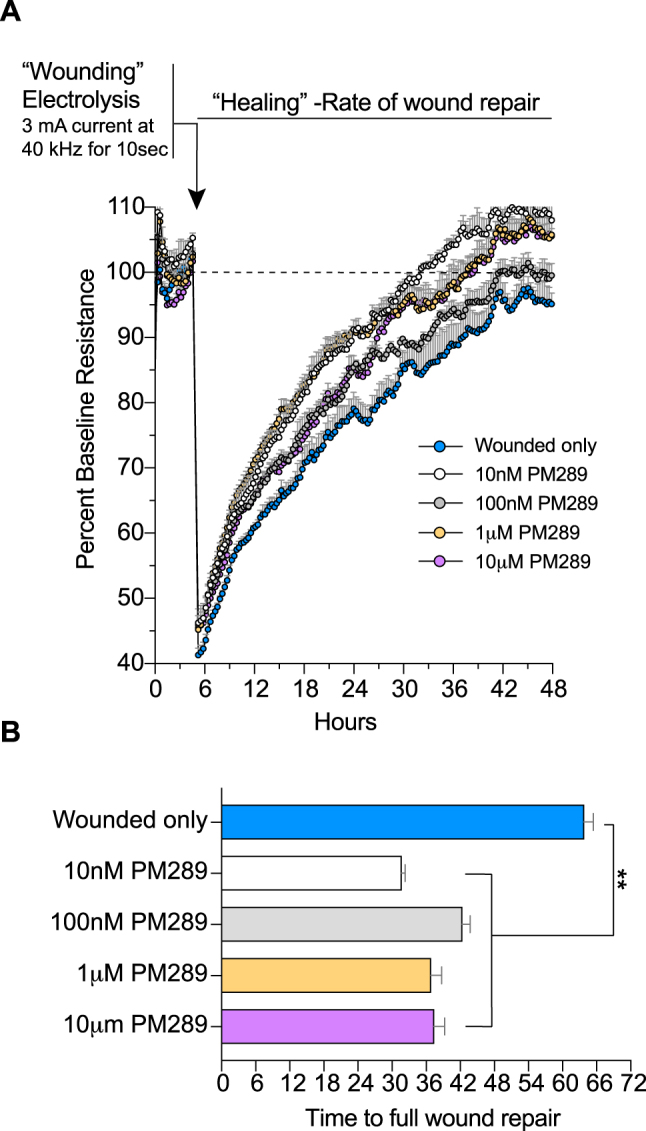
PM289 increases wound repair in brain endothelial cells. A. hCMEC/D3 cells were grown on gold electrodes until confluency. Cells over the gold were wounded by electrolysis with 3 mA current at 40 kHz for 10 s. Prior to wounding, cells were treated with concentrations of PM289 (10 nM, 100 nM, 1 μM and 10 μM). After wounding, the cells began to grow over the electrode until confluency was reached. B. The time to return to baseline resistance values was quantified for each condition. Cells treated with PM289 had a faster time to full would repair as compared to untreated cells. Each condition had n=4–8 replicates. **p<0.01.

To evaluate the effects of CB2R activation on endothelial repair, the following studies utilized an ECIS-based wound healing assay [33]. As before, hCMEC/D3 cells were plated on coated electrode arrays and allowed to reach confluency and stable barrier properties (based on their TEER values). Once confluent, the cells were treated with the novel CB2R agonist (PM289, 10 nM, 100 nM, 1 μM or 10 μM) for 5 h before applying the following wounding parameters: 60,000 Hz for 20 s at 1,400 µA. Figure 4 shows the results from continuous TEER monitoring in real-time before and after the wounding protocol was initiated.

Figure 4A shows real-time traces of normalized resistance. Wound induced an immediate reduction (approximately a 58 % drop) in normalized TEER values compared to baseline readings. TEER measurements were taken for the ensuing 48 h in which recovery, represented by a rise in TEER, can be observed. In wounded cells that had been treated with PM289, faster rates of healing were observed (F[4,10]=65.09, p<0.0001). Conversely, concurrent treatments with PM289 and SR144528 prior to wounding, did not exhibit faster rates of recovery (data not shown). As shown in Figure 4B, the wounded cells that had been treated with PM289, showed faster time to recovery. As can be seen in the graph, time to full recovery was accomplished maximally at 2.1 times faster with 10 nM PM289 compared to those without CB2R agonist. Other concentrations improved healing to full recovery by 1.5 times and 1.75 times faster for 100 nM and both 1 and 10 μM respectively. The benefits of CB2R activation (at least by PM289) on healing kinetics appear to display a bimodal effect since the lowest concentration of PM289 had a robust effect that then lessen and then strengthen as concentrations were increased. Neither PM289 nor SR144528 alone had an adverse effect on hCMEC/D3 cell TEER values (data not shown). Together, these data suggest that PM289 significantly enhances wound recovery in a CB2R dependent manner.

### CB2R activation in brain endothelial cells inhibits NFκB activation

**Figure 5:**
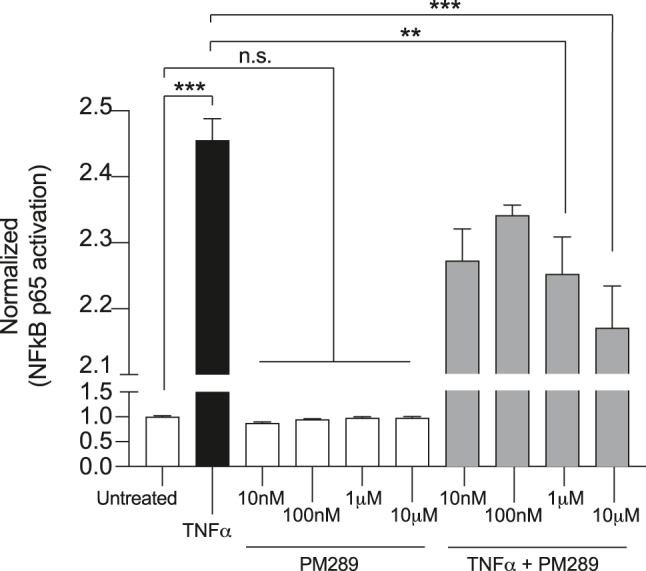
PM289 attenuates NFκB activation in response to TNFα. Cells were treated with TNFα with or without concentrations of PM289 (10 nM, 100 nM, 1 μM and 10 μM) for 24 h. Cell lysates were then analyzed for p65 subunit activity with the TransAM NFκB Activation Assay. TNFα increased p65 activity which was statistically decreased by PM289. All groups were performed in replicates of n=3. **p<0.01.

In an effort to arrive at a possible cell signaling explanation for how CB2R activation may mediate anti-inflammatory effects in brain endothelial cells, the activation of NFκB (p65) was investigated. As before, hCMEC/D3 cells were grown to confluency and treated with TNFα (100 ng/mL), PM289 (10 nM–10µM), or combinations thereof. After 24 h, treatments were removed, cells were washed and processed for analysis of NFκB using a transcription factor ELISA-based assay (Figure 5).

TNFα significantly increases NFκB (p65) activity over 2.4 fold above the untreated control. Cells treated together with TNFα and PM289 also exhibited increased NFκB p65 activity, but this increase was significantly lower than what was observed in cells treated only with TNFα (F[9,20]=374.7, p<0.0001). NFκB inhibition offers a possible explanation as to the anti-inflammatory action of CB2R activation in brain endothelial cells, suggesting that the binding sites for NFκB on the ICAM1 promoter may be less activated by CB2R.

## Discussion

The central hypothesis of the present research was to explore whether CB2R activation at the level of the brain endothelial cells would render vascular protective effects. To that end, the results herein illustrate that a novel and highly selective, chromenopyrazole based CB2R agonist, PM289, can regulate both the physical and immunological barrier functions of brain endothelial cells. Moreover, these data show for the first time in an experimental preclinical model of traumatic brain injury, significant upregulation in the CB2R gene expression at the level of the cerebral vasculature. The inducibility of CB2R begins as early as 4 h and increased expression is sustained up to 48 h. These results point to the important notion that CB2R is not only expressed at the brain endothelium but that it is also greatly upregulated during conditions of neuroinflammation. Importantly, the above offers therapeutic opportunities for targeting of CB2R to modulate inflammatory responses in manners which are both vascular and BBB protective. It is well accepted that BBB function can be improved by anti-inflammatory agents such as steroids [34] and other classes of immunomodulatory pharmacological compounds.

Previous studies have shown that brain vascular expression of CB2R and upregulation of the receptor can occur during neuroinflammatory conditions and models of sepsis [10]. The present data illustrates that these findings can also be expanded to experimental neurotrauma using the CCI-TBI model. CCI-TBI uses a controlled cortical impactor to deliver a blunt force injury to the brain of anesthetized mice which has long been used as a reproducible and standardized preclinical model of TBI [35]. The pathologic features of moderate severity CCI-TBI are congruent with the human condition that include neurodegeneration, neuroinflammation, and reactive astrogliosis. Furthermore, CCI-TBI in mice mimics cortical tissue loss, acute subdural hematoma, diffuse axonal injury, concussive syndromes, and BBB dysfunction that characterize the TBI pathology seen in humans. As with other neurological disorders and disease states, neurotrauma is accompanied by a massive inflammatory response due to cell injury and death [36]. It has been postulated that the induction of CB2R, like that which was observed here, may point towards its contribution in the resolution of the inflammatory response [37].

As such, subsequent experiments were conducted to assess the effects of inflammation and CB2R activation on brain endothelial cells. The response of the endocannabinoid system to inflammation has been extensively investigated. For example, peripheral and CNS immune cells modulate their expression of both CB1R and CB2R in response to pro-inflammatory stimuli [10, 38, 39]. It has been suggested that this upregulation is a part of the pathogenic response and necessary for the resolution of inflammation [10, 40]. While both CB1R and CB2R activation exhibit similar anti-inflammatory effects [10, 41], [42], [43], the association of CB1R with addictive drug properties precludes therapeutic strategies targeting this receptor. CB2R is minimally associated with psychotropic effects and is therefore a *de facto* treatment target for controlling inflammation. Notably, CB2R is purported to regulate inflammatory processes both within and exterior to the CNS [9, 44, 45]. Here, CB2R activation is achieved with the novel, potent chromenopyrazole based agonist, PM289. PM289 is an N2-ethyl and phenol-methyl substituted analog of the chromenopyrazole chemical backbone. With a Ki of 92.6 ± 17.1 nM, PM289 is approximately 32-fold more selective for CB2R over CB1R [23]. Exposure of brain endothelial cells to PM289 did not affect cell viability, even at the highest of concentrations used (Supplemental Figure 1), making this novel compound an ideal candidate for investigating vascular therapeutic effects of CB2R activation.

To that end, the human brain endothelial cell line, hCMEC/D3 was utilized as a model of the BBB. Using TNFα to induce physical barrier disruption and endothelial activation in hCMEC/D3 cells, the experiments herein show that PM289 prevents physical barrier disruption and regulates endothelial activation. Although neither of these drug effects fully restored what was observed in untreated/baseline cells, these findings support what has been observed in previous research with other CB2R agonists, namely JWH133 and O-1966. Notably, previous research utilized micromolar doses of JWH133 and O-1966 to induce an effect in their respective studies [16] whereas here, PM289 induced a cellular response over a range of doses from 10 nM to 10 µM. Moreover, a direct comparison between PM289 and JWH133 showed an equal concentration of PM289 abrogated TNFα-induced paracellular permeability to small molecule diffusion to a greater degree than that which was observed following JWH133 treatment (Supplemental Figure 2). Interestingly, co-administration of PM289 and the CB2R antagonist, SR144528 reversed the effects of PM289, but only at select doses suggesting that there may be additional biological targets responsible for the changes induced by PM289, especially in the micromolar range. Alternatively, early research with SR144528 indicated this substance has both antagonist and inverse agonist activity in relation to CB2R [44]. Although the cellular responses investigated in the present experiments did not reveal SR144528 alone to have an effect, little is known regarding brain endothelial CB2R, so there remains the possibility that the antagonist has yet undiscovered effects in this cell type.

Following experiments to address the therapeutic potential of PM289 and endothelial CB2R, assays were conducted to investigate the molecular signaling cascades which may be governed by CB2R. The assay utilizes consensus-binding sites on oligos for the NFκB (p65) transcription factor immobilized in multi-well plates. Active NFκB in cell lysates bind to the oligos which is then detected with NFκB specific antibodies conjugated to enzymes that convert a chemiluminescent substate. NFκB is a known regulator of the endothelial activation response and principally regulates endothelial expression of adhesion molecules, among other inflammatory processes [45]. The present data shows the expected activation of the NFκB p65/RelA subunit when hCMEC/D3 cells were treated with TNFα. Interestingly, concurrent treatment of cells with TNFα and PM289 did not significantly reduce p65 activation below what was observed with TNFα alone except at the 1 and 10 µM doses. The reduction in NFκB offers an explanation as to the effects of CB2R signaling on induced ICAM1 expression by pro-inflammatory cytokines. The studies here show that the enhanced expression of ICAM1 by TNFα is significantly attenuated by CB2R activation. It is well stablished that a κB-site exists within the promoter region for the ICAM1 gene [46]. Thus, it is reasonable to consider that the partial inhibition in ICAM1 upregulation by CB2R activation, in cells activated by TNFα and possibly other pro-inflammatory cytokines, is due to CB2R effects on p65. Interestingly, the κB-site in the ICAM1 promoter can also bind cRel, another member of the NFκB family of transcription factors. Perhaps future studies could be performed to characterize whether CB2R signaling targets the inhibition of other NFκB family members.

Another essential aspect of vascular protection includes the ability for an intervention to promote regeneration and reendothelialization of damaged or injured vasculature. Anatomical disruption to the endothelium is part of the pathology of brain injury, stroke, cerebral vascular disease and many more conditions that cause neurological impairment. The results of the studies presented here, show that CB2R activation by the potent CB2R agonist PM289, drastically enhances wound healing of endothelial cells. The results shown are from an electroporation/electrolysis-based wounding assay, that is highly precise, reproducible and analytical [33]. The advantage of the assay is that the proliferation and migration of cells can be monitored in real-time by measuring TEER. These analyses are also the first to provide evidence that CB2R activation in brain endothelial cells accelerates endothelial cell recovery. How wound repair is mediated by CB2R is unknown. Future studies will focus on whether CB2R promotes angiogenic pathways, stimulates growth factor production or whether any effects on cell migration can be seen.

Importantly, the primary goal of the present research was to further explore and elucidate the role of CB2R on endothelial cells. While neuroinflammatory responses affect all brain cell types, the contribution of the cerebral vasculature to resolution of inflammation represents a novel and interesting target for future treatment development. Notably, this type of therapeutic development could work in conjunction with treatments directed to other cell types. For example, the anti-inflammatory role of CB2R within the brain is well established insofar as activation is known to reduce TNFα production and attenuate ICAM1 expression [47–49]. Whereas these findings were presented in relation to immune cells and whole-brain tissue, respectively, the results of the present work are specific to cerebral endothelial cells. Taken together, these findings demonstrate how CB2R activation could be broadly beneficial in relation to neuroinflammatory pathologies via mechanisms which target multiple cell types and pathways.

In summary, the present research highlights the inducible nature of endothelial CB2R and extends previous findings to experimental models of TBI. Moreover, these data demonstrate the vascular protective effects of a novel, chromenopyrazole based CB2R agonist indicating specifically that activation of endothelial CB2R regulates both physical and immunological barrier properties in human brain endothelial cells which also promotes wound healing. These data also show for the first time that endothelial CB2R can inhibit NFκB activation. Future research will be necessary to determine the signaling mechanisms responsible for the accelerated endothelial repair that was observed. Overall, these findings further support the notion that the anti-inflammatory effects of CB2R in the brain endothelium could be a novel therapeutic avenue for attenuating vascular inflammatory responses and improving BBB integrity during neuroinflammatory conditions.

## Supplementary Material

Supplementary Material Details

Supplementary Material Details
