# Supplementary figures and images for: Activation of CB2R by synthetic CB2R agonist, PM289, improves brain endothelial barrier properties, decreases inflammatory response and enhances endothelial repair
Source: NeuroImmune Pharm Ther. 2023 Oct 16;2(4):387–400. doi: 10.1515/nipt-2023-0016 (PMC10726734; doi:10.1515/nipt-2023-0016)

Supplemental Figure 1

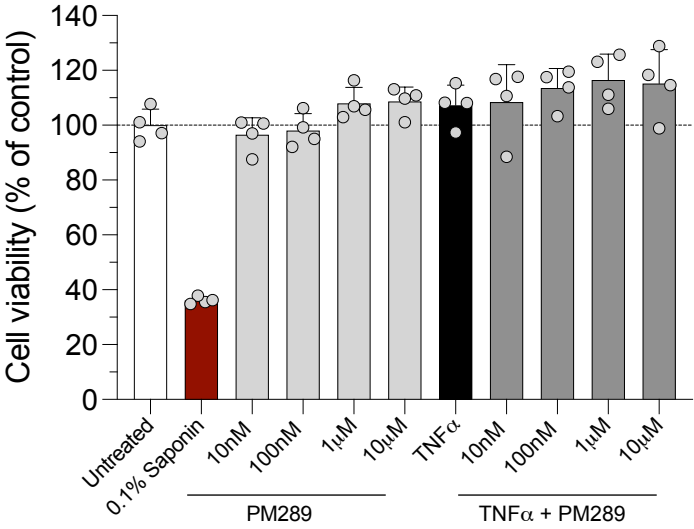

Supplement: Supplementary file 1 — Supplementary Material Details [file j_nipt-2023-0016_suppl_001.pdf]

Supplemental Figure 2

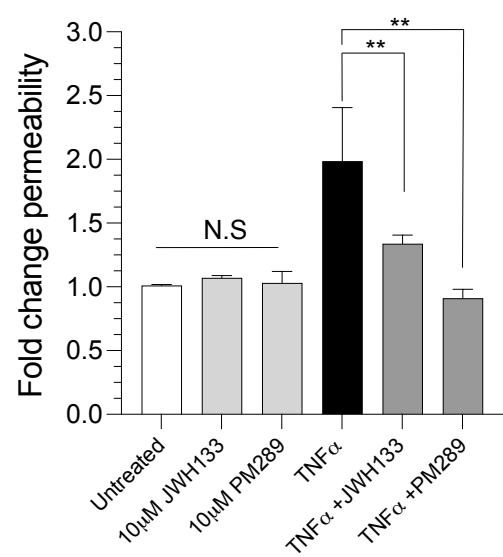

Supplement: Supplementary file 2 — Supplementary Material Details [file j_nipt-2023-0016_suppl_002.pdf]
